# Middle Frontal Gyrus and Area 55b: Perioperative Mapping and Language Outcomes

**DOI:** 10.3389/fneur.2021.646075

**Published:** 2021-03-10

**Authors:** Sally Rosario Hazem, Mariam Awan, Jose Pedro Lavrador, Sabina Patel, Hilary Margaret Wren, Oeslle Lucena, Carla Semedo, Hassna Irzan, Andrew Melbourne, Sebastien Ourselin, Jonathan Shapey, Ahilan Kailaya-Vasan, Richard Gullan, Keyoumars Ashkan, Ranjeev Bhangoo, Francesco Vergani

**Affiliations:** ^1^Department of Neurosurgery, King's College Hospital National Health Service Foundation Trust, London, United Kingdom; ^2^King's Neuro Lab, Department of Neurosurgery, King's College Hospital National Health Service Foundation Trust, London, United Kingdom; ^3^School of Biomedical Engineering and Imaging Sciences, King's College London, London, United Kingdom; ^4^Department of Medical Physics and Biomedical Engineering, University College London, London, United Kingdom

**Keywords:** area 55b, language mapping, speech arrest, perioperative mapping, DTI, TMS, language network, nTMS

## Abstract

**Background:** The simplistic approaches to language circuits are continuously challenged by new findings in brain structure and connectivity. The posterior middle frontal gyrus and area 55b (pFMG/area55b), in particular, has gained a renewed interest in the overall language network.

**Methods:** This is a retrospective single-center cohort study of patients who have undergone awake craniotomy for tumor resection. Navigated transcranial magnetic simulation (nTMS), tractography, and intraoperative findings were correlated with language outcomes.

**Results:** Sixty-five awake craniotomies were performed between 2012 and 2020, and 24 patients were included. nTMS elicited 42 positive responses, 76.2% in the inferior frontal gyrus (IFG), and hesitation was the most common error (71.4%). In the pMFG/area55b, there were seven positive errors (five hesitations and two phonemic errors). This area had the highest positive predictive value (43.0%), negative predictive value (98.3%), sensitivity (50.0%), and specificity (99.0%) among all the frontal gyri. Intraoperatively, there were 33 cortical positive responses—two (6.0%) in the superior frontal gyrus (SFG), 15 (45.5%) in the MFG, and 16 (48.5%) in the IFG. A total of 29 subcortical positive responses were elicited−21 in the deep IFG–MFG gyri and eight in the deep SFG–MFG gyri. The most common errors identified were speech arrest at the cortical level (20 responses−13 in the IFG and seven in the MFG) and anomia at the subcortical level (nine patients—eight in the deep IFG–MFG and one in the deep MFG–SFG). Moreover, 83.3% of patients had a transitory deterioration of language after surgery, mainly in the expressive component (*p* = 0.03). An increased number of gyri with intraoperative positive responses were related with better preoperative (*p* = 0.037) and worse postoperative (*p* = 0.029) outcomes. The involvement of the SFG–MFG subcortical area was related with worse language outcomes (*p* = 0.037). Positive nTMS mapping in the IFG was associated with a better preoperative language outcome (*p* = 0.017), relating to a better performance in the expressive component, while positive mapping in the MFG was related to a worse preoperative receptive component of language (*p* = 0.031).

**Conclusion:** This case series suggests that the posterior middle frontal gyrus, including area 55b, is an important integration cortical hub for both dorsal and ventral streams of language.

## Introduction

Previous models of parcellation of the cerebral cortex have been proposed based on cytoarchitectonic (1, 2), myeloarchitectonic (3, 4), or functional characteristics of the different cerebral cortical areas (5, 6).

More recently, a new mapping of the human cortex has been described, using a multi-modal gradient-based parcellation approach (7). One of the novelties of this approach has been the identification of new cortical areas with a distinctive myelo/cytoarchitectonic and functional profile. A particularly interesting region is the frontal area 55b. Initially noted by Hopf in 1956 (4), this area is located at the posterior aspect of the middle frontal gyrus (MFG) and is delimited by the frontal eye field (FEF) superiorly, the premotor eye field (PEF) inferiorly, the primary motor cortex and the ventral motor cortex posteriorly, and by the prefrontal areas anteriorly (7, 8). Area 55b appears to be lightly myelinated and lies between moderately myelinated areas (i.e., FEF above and PEF below) and anteriorly to heavily myelinated areas (i.e., primary motor cortex). It has been described to be involved in various language production tasks and fluency of speech (7, 9, 10). These findings are responsible for the renewed interest in the contribution of the posterior MFG to the overall language network.

Techniques of brain mapping that have evolved to increase the extent and safety of tumor resection in eloquent areas of the brain (11) have the unique advantage of testing different functions of specific cortical areas and networks at the individual level (12). Direct electrical stimulation (DES) at the cortical and subcortical levels is the gold standard for intraoperative mapping, defining the functional borders of resection in glioma surgery (13–15). In addition, navigated transcranial magnetic stimulation (nTMS) has emerged over the past decade as a useful adjunct for the preoperative mapping of motor (16–21) and language (22–27) areas of the brain.

In the present paper, we reviewed the results obtained by combining DES and nTMS in the functional assessment of the middle frontal gyrus and area 55b in a series of patients undergoing awake surgery for brain tumors. In addition, we evaluated the potential relationship between preoperative and intraoperative language mapping and between the assessment of language performed prior to and following surgery, with a view to assess the relative contribution of the MFG on the language outcome. The preoperative and intraoperative findings are reviewed, and the potential role of these areas as part of the language network is discussed.

## Materials and Methods

This is a retrospective single-center cohort study of non-consecutive patients admitted with language eloquent tumors for surgical treatment from January 2012 to January 2020. The inclusion criteria for the current study were age above 18 years old, awake craniotomy with DES for language mapping, and a tumor located in the dominant frontal lobe. Hemispheric dominance was assessed with the Edinburgh Handedness Inventory scale. The exclusion criteria included failed awake craniotomy and awake craniotomy for non-language mapping purposes.

### Language Assessment

The preoperative and postoperative assessments were performed using the Sheffield Aphasia Screening Test for Acquired Language Disorder (SST) (28). This test was applied by the same speech and language therapist responsible for the intraoperative language testing. The patients were interviewed pre- and postoperatively to assess their communication abilities in conversational speech. Subtle subjective changes pertaining to comprehension, speech, reading, or writing abilities affecting daily living were evaluated. Where relevant, additional subtests were administered from the Mount Wilga Higher Level Language Test (29). The language errors were divided into speech arrest, hesitation, fluency disturbance, repetition disturbance, semantic paraphasia, and anomia.

### Intraoperative Mapping

An asleep–awake–asleep craniotomy was performed in all the included patients. Low-frequency intraoperative stimulation according to the Penfield technique (30) was performed. Then, 50-Hz biphasic square wave pulses of 1-ms duration were applied using a constant current stimulator (ISIS Neurostimulator; Inomed Medizintechnik GmbH). The current threshold used for brain mapping was the minimal current responsible for speech arrest during the counting task (two out of three attempts) or the highest current non-responsible for after-discharges. The exposed cortical area was mapped with one stimulation area every 2–3 cm at least three times per language task. The selection of the intraoperative tasks were performed according to the Dutch Linguistic Intra-operative Protocol (31) and the Verb and Noun Test for Peri-Operative Testing (32).

Positive and negative responses were recorded, indicating the presence and absence of language errors elicited by DES respectively. After a speech arrest was identified and the threshold was established, the entire exposed cortical area of the frontal lobe was mapped with object naming and action naming in both present and past tenses. At the subcortical level, repetition tasks were used for mapping the arcuate fasciculus (AF), sentence completion for the fronto-aslant tract (FAT), and semantic odd-one-out and object naming for the inferior fronto-occipital fasciculus (IFOF). The positive responses—two out of three attempts—with induced speech deficit in the absence of after-discharges on electrocorticography were documented with intraoperative pictures.

### Navigated Transcranial Magnetic Stimulation

Preoperative mapping with nTMS has been used at our center since 2016 as an adjunct to intraoperative DES mapping. Whenever available, data from nTMS were included in the analysis.

Language mapping was performed following resting motor threshold determination (33). A set series of pre-designed images were presented to the patient for baseline assessment in two consecutive rounds: object naming, action naming in the present tense, and action naming in the past tense (32). The inter-picture interval was set to 2,500 ms, and the display time (DT) varied between 500 and 1,000 ms, which was dependent on a patient's ability. Images that were incorrectly described or with hesitation were excluded from the final exam. An offline analysis was performed, comparing the stimulation assessment to the baseline assessment and identifying any changes in language function during the exam. Language errors were classified into distinct categories (hesitation, expressive, semantic, anomia, arrest, other, and no errors).

The intraoperative pictures of positive stimulation sites collected during intraoperative mapping were included in the preoperative MRI studies (T1-weighted images after gadolinium injection) by comparing the anatomical landmarks (i.e., sulci and gyri) of the single pictures with the axial brain volumetric images and reformatted sagittal/coronal images (33). It was therefore possible to correlate the positive intraoperative sites with nTMS mapping, allowing for the calculation of TMS specificity, sensitivity, and positive and negative predictive values (PPV and NPV, respectively).

### Statistical Analysis

STATA 13.0 software was used for statistical analysis. Regression techniques were performed to compare the language outcomes—screening test for acquired language disorder (SST) and its receptive and expressive subdivisions—with the number of gyri and the main subcortical areas infiltrated by the tumor. A *p*-value < 0.05 was considered as significant.

## Results

**Figure 1:**
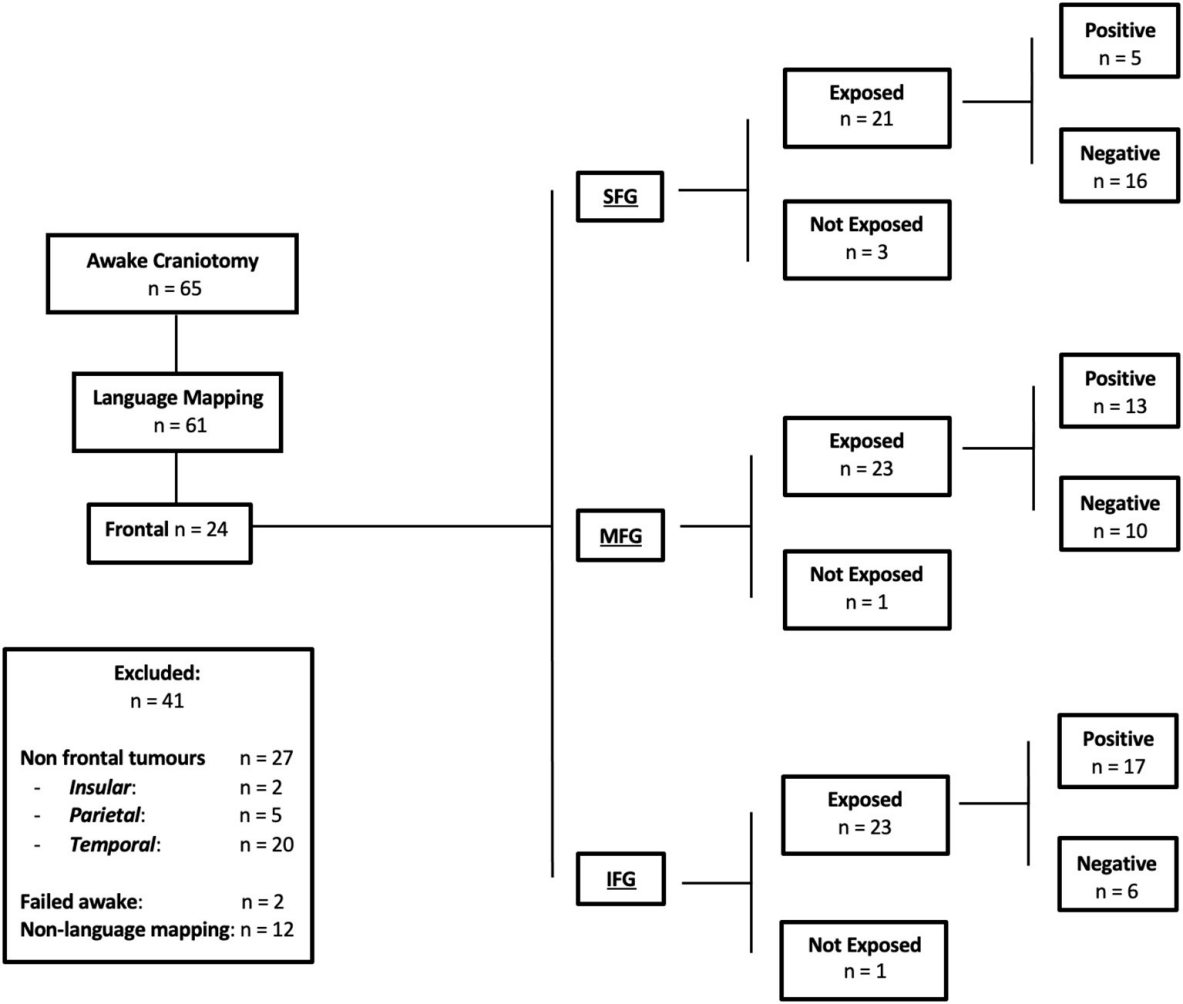
Flow chart of the present study, detailing the inclusion and exclusion criteria. Positive responses indicate language errors elicited by direct electric stimulation. Negative responses indicate no language errors elicited by direct electric stimulation. Exposure indicates the specific frontal gyrus exposed during craniotomy.

Sixty-five awake craniotomies were performed between 2012 and 2020. There were 24 (36.9%) frontal tumors that constitute the object of the current study. The demographics showed an even distribution between males and females, with a mean age of 47 ± 13.8 years old (standard deviation). The majority of the included patients had left-sided tumors (22, 91.6%). Meanwhile, 19 (79.1%) patients presented with a new-onset seizure; two (8.3%) had a motor deficit, and two (8.3%) presented with a language deficit. The vast majority of patients (20, 83.4%) presented with a performance status of 0. High-grade gliomas were prevalent in this series [71% World Health Organization (WHO) grade III and IV]. Isocitrate dehydrogenase was positive in 10 (41.6%) tumors, whereas 1p/19q co-deletion was present in nine (37.5%) oligodendrogliomas. Figure 1 and Table 1 summarize the patients' characteristics.

**Table 1:** Demographics of the included subjects along with the classification of tumors based on histology, grade, and biomarkers.

**Subject demographics and tumor classification**	
**Sex**	
Female	12 (50%)
Male	12 (50%)
**Age group**	
18–24	1 (4.1%)
25–34	5 (20.8%)
35–44	5 (20.8%)
45–54	5 (20.8%)
55–64	6 (25%)
65–74	2 (8.3%)
**Presentation**	
Motor deficit	2 (8.3%)
Language deficit	2 (8.3%)
Cognitive deficit	1 (4.2%)
Seizure	19 (79.2%)
**Tumor laterality**	
Right	2 (8.3%)
Left	22 (91.6%)
**Histology**	
Anaplastic Astrocytoma	3 (12.5%)
Anaplastic Oligodendroglioma	6 (25%)
Diffuse Astrocytoma	4 (16.7%)
Glioblastoma Multiforme	5 (21%)
Glioneuronal Tumor	1 (4%)
High Grade Glioma	2 (8.3%)
Low Grade Glioma	3 (12.5%)
Oligodendroglioma	3 (12.5%)
**WHO grading**	
I	0 (0%)
II	3 (12.5%)
II	4 (16.7%)
III	12 (50%)
IV	5 (21%)
**Tumor marker**	
***IDH***	
Positive	10 (41.6%)
Negative	7 (29.1%)
Mutant	6 (25%)
Wildtype	1 (4.2%)
***1p/19q***	
Co deletion	9 (37.5%)
No deletion	2 (8.3%)
19q deletion	1 (4.2%)
***N/A***	12 (50%)

### Preoperative Language Assessment

The average preoperative SST was 18.9/20. The average receptive language skill score was 8.1/9, and the average expressive language skill score was 10.7/11 (seven patients were excluded due to incomplete assessments). From the SST, the most frequent receptive errors were auditory semantic differentiation and the ability to fully comprehend a paragraph-level narrative. Expressive errors were rare, but when present, the most frequent error was the ability to provide word definitions. The SST does not measure phonemic ability, but no patient displayed any marked phonemic errors in conversation preoperatively (Table 2).

**Table 2:** A comprehensive table detailing the navigated transcranial magnetic simulation language errors, intraoperative stimulation positive responses, a comparison of language pathway error, Sheffield Aphasia Screening Test (SST) for Acquired Language Disorder assessments pre- and postoperatively, changes in SST score, and a comprehensive speech and language therapy pre-, intra-, and postoperative assessment.

**ID**	**nTMS Language Error**	**Intra-operative mapping response**	**Sheffield screening test (SST)**			**Speech and language therapist (SLT) assessment**		
			**Preoperative**	**Postoperative**	**Difference in scores**	**Preoperative**	**Intraoperative**	**Postoperative**
1	*SFG:* None *MFG*: Stutter (1) *IFG*: Hesitation (3)	SFG-MFG	Total: Spanish: 18/20 *English*: 13/20	None *2 years post op:* 12/20	Total: −6	Mild receptive and expressive dysphasia Semantic errors in Spanish Action and object naming difficulties	No language errors in English or Spanish	Mild receptive and expressive dysphasia Increase in word finding difficulties compared to pre op (in both languages)
2	*SFG:* None *MFG*: Word finding difficulty (1) *IFG*: None	SFG-MFG	Total: 15/20 Receptive 6/9 Expressive 9/11	Total: 9/20 Receptive 4/9 Expressive 5/11	Total: −6 Receptive −2 Expressive −4	No obvious dysphasia Difficulties more likely to be due to English as additional Action and object naming difficulties	Semantic errors, hesitation in Farsi for object naming. End of awake period: difficulty with object naming in both languages More difficulty in first language (Farsi) than in English	Moderate dysphasia in English Word finding difficulties (semantic errors) and perseveration in English (5 days post op)
3	*SFG:* None *MFG*: None *IFG:* Hesitation, Apraxia, Semantic Anomia (13)	IFG-MFG	Total: 20/20	*2 days post op:* unable to participate *5 days post op:* Total: 14/20 Receptive 8/9 Expressive 6/11	Total: −6 Receptive −1 Expressive −5	No overt dysphasia in conversation	Hesitation and semantic error (object naming, action naming) with 2 perseveration errors in object naming	Mild/moderate dysphasia Difficulty with complex reading tasks Hesitancy Word finding difficulty
4	*SFG:* None *MFG*: None *IFG:* None	SFG-MFG	Total: 19/20 Receptive 8/9 Expressive 11/11	Total: 2/20 Receptive 2/9, test abandoned as too difficult	Total: −17 Receptive −6	No communication and language dysfunction	Semantic, phonemic, reading, and fluency errors	Moderate expressive and receptive dysphasia Fluency errors and difficulties with semantic tasks Spontaneous speech notably easier than when asked direct questions
5	*SFG:* None *MFG*: None *IFG:* Not performed	SFG-MFG	Total: 19/20 Receptive 8/9 Expressive 11/11	Unable to complete SST *7 days post op:* Total: 13/20 Receptive 8/9 Expressive 5/11	Total: −6 Receptive 0 Expressive −6	Semantic errors	Semantic errors SMA-like syndrome No automatic speech, counting errors	Semantic and phonemic errors Moderate expressive aphasia
6	Not performed	SFG-MFG	Total: 20/20	Total: 18/20	Total: −2	Difficulty following complex commands Phonemic errors in conversation reported by family but not seen in clinic	No difficulties with repetition, object or verb naming	*5 days post op*: mild receptive and severe expressive dysphasia with likely overlay of verbal dyspraxia *1 month post op:* mild expressive dysphasia Semantic word finding difficulties in conversation and/or difficulties with grammatical structure
7	*SFG:* Hesitation (1) *MFG*: Hesitation (1) *IFG:* Word formation (1)	SFG-MFG	Total: 19/20 Receptive 8/9 Expressive 11/11	*5 days post op* unable to complete: severe expressive dysphasia, SMA initiation difficulties. *7 days post op:* Total: 13/20 Receptive 7/9 Expressive 6/11	Total: −6 Receptive −1 Expressive −5	No overt dysphasia in conversation.	Speech arrest 2 semantic errors 1 hesitation error (all in verb naming) 2 sentence completion errors	Mild receptive with moderate expressive dysphasia Semantic difficulties with additional difficulties initiating speech
8	*SFG:* None *MFG*: None *IFG:* Hesitation (6)	IFG-MFG	Total: 18/20 Receptive 8/9 Expressive 10/11	Total: 18/20 Receptive 8/9 Expressive 10/11	Total: 0 Receptive 0 Expressive 0	No overt dysphasia in conversation but semantic difficulties in testing.	Semantic, hesitation, fluency errors.	Mild dysphasia - mild word finding difficulties in conversation - using circumlocution to good effect
9	*SFG:* Anomia (2) *MFG*: Hesitation (2) *IFG:* None	SFG-MFG	Total: 18/20 Receptive 7/9 Expressive 11/11	Total: 9/20 Receptive 2/9 Expressive 7/11 (cognitive overlay)	Total: −9 Receptive −5 Expressive −4	No overt difficulties, occasional hesitations Semantic errors	Word finding difficulties in conversation and object naming	Mild dysarthria, moderate dysphasia with phonemic errors in conversation Cognitive communication difficulties
10	*SFG:* None *MFG*: None *IFG:* Hesitation (6)	IFG-MFG	Total: 20/20	Total: 14/20 Receptive 9/9 Expressive 5/11	Total: −6 Receptive 0 Expressive −6	No overt dysphasia	2 phonemic errors in mapping	Mild to moderate expressive dysphasia Motor planning difficulties
11	*SFG:* None *MFG*: Hesitation (1) *IFG:* None	IFG-MFG	Total: 17/20 Receptive 6/9 Expressive 11/11	Total: 20/20	Total: +3 Receptive +3 Expressive 0	No communication difficulties in conversation	Imprecise articulation/dysarthric errors in mapping (2) and in cortical resection	No obvious dysphasia
12	Not performed	None	Not performed	Not performed	Unable to determine	No obvious dysphasia in first language (Polish)	Perseveration and word finding difficulties during resection	No data available
13	Not performed	IFG-MFG	Total: 19/20 Receptive 8/9 Expressive 11/11	Total: 14/20 Receptive 6/9 Expressive 8/11	Total: −5 Receptive −2 Expressive −3	Higher level word finding difficulties	4 phonemic errors in mapping, 1x phonemic error and 1x hesitation in resection	Mild receptive and expressive dysphasia Word finding difficulties more evident in testing than in conversation
14	*SFG:* None *MFG*: Hesitation (1) *IFG:* Hesitation, Anomia (2)	IFG-MFG	Total: 20/20 Receptive 9/9 Expressive 11/11	Total: 15/20 Receptive 7/9 Expressive 8/11	Total: −5 Receptive −2 Expressive −3	Initial difficulty in recalling details	Phonemic errors noted in mapping (2 separate areas) Self-correcting phonemic errors in conversation during resection 1 phonemic error at final testing at the end of resection	Mild receptive and expressive dysphasia Improvements noted in verbal sequencing compared to immediately after previous surgery
15	*SFG:* None *MFG*: None *IFG:* Hesitation, Dysarthria, Semantic (4)	IFG-MFG	Total: 20/20	Total: 18/20 Receptive 8/9 Expressive 10/11 (phonemic errors not significant to impact on score)	Total: −2 Receptive −1 Expressive−1	No communication difficulties	Action naming, 1 clear speech arrest in lead up phrase Object naming, phonemic difficulty during mapping, resection of arcuate fasciculus Slurred speech at the end of resection	Mild word finding difficulties with lower frequency nouns Mildly reduced associated naming Mild-moderate verbal apraxia, mild dysarthria and mild expressive dysphasia
16	Not performed	None	Not performed	Not performed	Unable to determine	No communication difficulties reported	No data available	Mild receptive and moderate-severe expressive dysphasia Unable to speak in phrases or sentences, occasional word, using Yes/No
17	Not performed	None	Not performed	Not performed	Unable to determine	All normal except planning (constructing sentences from given words) Mildly impaired in efficiency, auditory memory, auditory comprehension and numeracy	Prompting for biological information, one-word answers, Counting: perseveration at number 7 Visual and semantic errors on picture naming Speech arrest	*2 days post op*: moderate receptive and expressive dysphasia Semantic and phonemic errors Speech slow and effortful *5 days post op:* Mild/moderate dysphasia but still with phonemic and semantic errors
18	Not performed	None	Not performed	Not performed	Unable to determine	No overt dysphasia Mild difficulties with planning for sentence construction and in planning for sequencing on Mount Wilga higher level language tasks Some word finding difficulties previous to taking steroids	Minor visual and semantic errors Speech arrest very obvious during stimulation when counting 1–10 during first half of testing No obvious dysphasia in intra-operative testing. At end of testing, able to name single object pictures, describe pictures, repeat words and participate in conversation	No obvious dysphasia in conversation or Brisbane Language screen
19	Not performed	None	Not performed	Not performed	Unable to determine	No difficulties communicating in conversation Mild higher-level language difficulties in Mount Wilga tests (7/10 in auditory comprehension and recall questions, difficulty with jumbled sentences task) No difficulty with planning tasks.	No communication errors	Mild dysphasia Word finding difficulties in conversation (phonemic and semantic) and difficulties organizing sentences within a narrative
20	*SFG:* None *MFG*: None *IFG*: None	SFG-MFG	Total: 20/20	*Post op*: Total: 0/20 *Post op week 1*: Total: 8/20 *Post op week 2*: Total: 14/20 Receptive 9/9 Expressive 5/11	Total: −6 Receptive 0 Expressive −6	No difficulties in communication	No issues with naming objects and actions Possible hesitation with lower frequency items Later stages of resection, able to name intermittently, preservation and mild semantic errors noted At the end of resection, unable to name or repeat or count to 10	Severe expressive and receptive dysphasia Non-verbal post-op Overlay of difficulties initiating speech - at times these severely impact on patient's ability to make self understood
21	Not performed	SFG-MFG	Total: 19/20 Receptive 8/9 Expressive 11/11	Total: 20/20Receptive 9/9 Expressive 11/11	Total: +1 Receptive +1 Expressive 0	No communication difficulties	No communication errors but drowsy	No obvious dysphasia or difficulties in short conversation
22	Not performed	None	Not performed	Not performed	Unable to determine	Mild difficulty with written calculation and planning sentence construction	Periods of motor speech and naming deficits during mapping and surgery. At end of SLT assessment: -Decreased spontaneous verbal output -Producing automatics and single words/short phrases to sentence closure tasks	*2 days post op*: severe expressive dysphasia *5 days post op:* mild receptive with moderate expressive dysphasia Dyspraxia of speech, comprehending complex info during conversations.
23	*SFG:* None *MFG*: None *IFG:* Hesitation, Anomia (2)	IFG-MFG	Total: 18/20 Receptive 8/9 Expressive 10/11	Total: 16/20 Receptive 7/9 Expressive 9/11	Total: −2 Receptive −1 Expressive−1	Some higher-level language difficulties apparent	Minor difficulty with repetition during subcortical mapping Intermittent repetition, naming, and spontaneous speech during resection No difficulties with spontaneous speech at the end of surgery; able to answer direct questions, name high frequency objects and 9/10 on repetition tasks but fatigued	Mild dysphasia and higher level language difficulties likely linked to ability to retain and organize information
24	*SFG:* None *MFG*: None *IFG:* Language reversion to French (1)	SFG-MFG IFG-MFG	Total: 20/20	Total: 13/20 Receptive 6/9 Expressive 7/11	Total: −7 Receptive −3 Expressive −4	Mild to moderate impairment in verbal explanation. Moderate impairment in sentence construction and understanding inferential information, this is likely akin to mild dysphasia though EAL (English as an Additional Language)	Semantic errors in initial mapping, too drowsy to comment on phonemic errors	Mild/moderate receptive and expressive dysphasia Able to answer basic questions in conversation but difficulty with longer explanations: likely combination of cognitive communication difficulty and dysphasia

### Preoperative nTMS Assessment

nTMS was performed in 14 (58.3%) patients with frontal tumors (1,844 stimulations distributed across the gyri as follows: SFG−172, MFG−428, IFG−1,244), and positive responses were elicited in 12 patients (85.7%), with a total of 42 positive responses: three responses in the SFG (one hesitation and two anomic errors); seven responses in the MFG (five hesitations and two phonemic errors); 32 responses in the IFG (24 hesitations, six phonemic, and two semantic errors). This shows a preferential distribution of the stimulations in the IFG, particularly given the likelihood of positive responses in the area of the frontal operculum.

**Figure 2:**
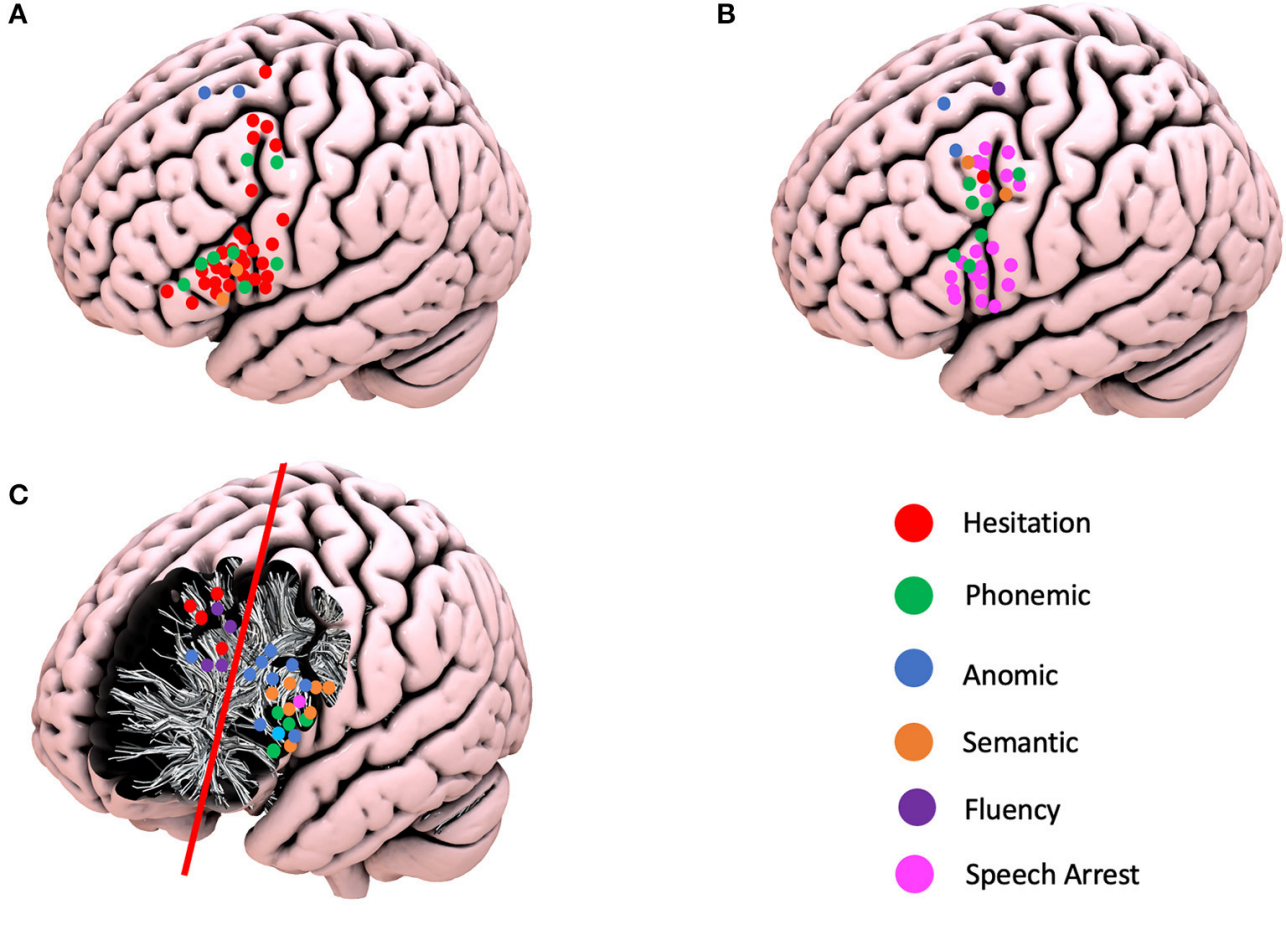
Schematic representation of the positive navigated transcranial magnetic simulation responses, intraoperative cortical responses, and positive subcortical responses according to the different language errors. **(A)** Positive TMS responses. **(B)** Positive cortical intraoperative responses. **(C)** Positive subcortical intraoperative responses.

Overall, the nTMS had a PPV of 31.0%, a NPV of 97.8%, sensitivity of 45.7%, and specificity of 98.3%. When the gyri were compared, the MFG had the highest PPV (43.0% vs. IFG−31.25% and SFG−0%), NPV (98.3% vs. IFG−97.8% and SFG−95.9%), sensitivity (50.0% vs. IFG−47.1%, and SFG−30.0%), and specificity (99.0% vs. IFG−98.2% and SFG−98.1%) (Table 2, Figure 2, and Supplementary Video 1).

**Figure 3:**
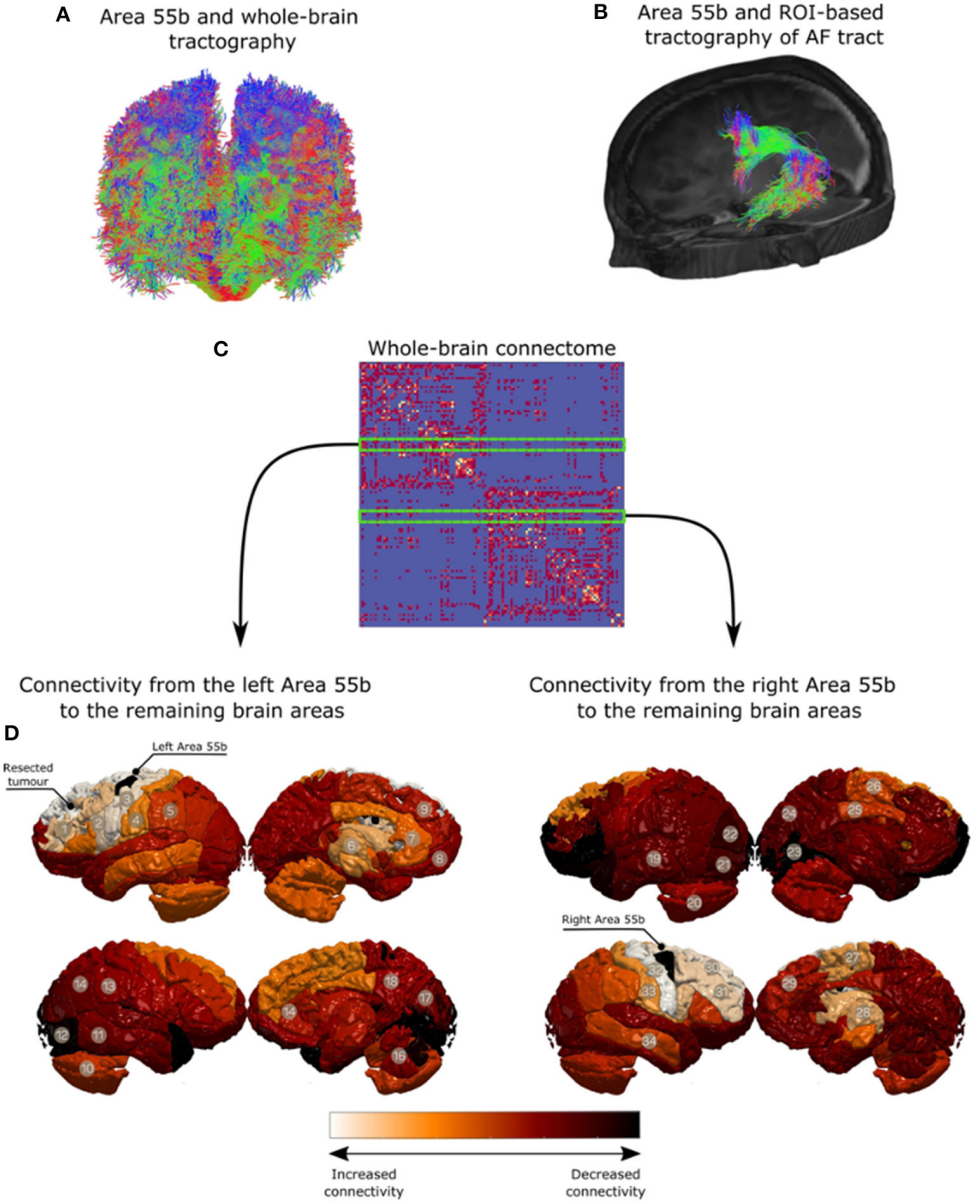
Qualitative analysis for region of interest-based tractography of the arcuate fasciculus (AF). The middle frontal gyrus region is in red, where the streamlines from the AF terminate. **(A)** Area 55b and whole-brain tractography. **(B)** Area 55b and ROI-based tractography of AF tract. **(C)** Whole-brain connectome. **(D)** Connectivity from the left Area 55b to the remaining brain areas. **(E)** Connectivity from the right Area 55b to the remaining brain areas.

A preoperative structural connectome analysis performed in three patients with positive responses in the area 55b showed the strongest connectivity of this area not only with the primary motor cortex but as well as with the supplementary motor area, inferior frontal gyrus, and anterior aspect of the MFG (Figure 3). Positive nTMS mapping in the inferior frontal gyrus was related with a better preoperative overall SST score (*p* = 0.017) due to a better receptive component (*p* = 0.001). Positive nTMS mapping for the posterior MFG/area 55b was related with a worse receptive preoperative component of the SST (*p* = 0.031), but with no expression in the overall score (*p* = 0.059) (Table 3).

**Table 3:** Correlation of navigated transcranial magnetic simulation responses per gyrus and the preoperative language assessment (Sheffield aphasia screening test for acquired language disorder).

	**Coef**.	**95%CI**	***p*-value**
**nTMS IFG**			
SST	0.21 ± 0.7	(0.05–0.38)	0.017
Receptive	0.35 ± 0.08	(0.18–0.53)	0.001
Expressive	0.22 ± 0.25	(−0.35–0.78)	0.407
**nTMS MFG**			
SST	−0.19 ± 0.09	(−0.38–0.01)	0.059
Receptive	−0.29 ± 0.11	(−0.54 to −0.03)	0.031
Expressive	−0.15 ± 0.26	(−0.75–0.45)	0.579
**nTMS SFG**			
SST	−0.01 ± 0.8	(−0.19–0.16)	0.877
Receptive	−0.07 ± 0.11	(−0.32–0.18)	0.538
Expressive	0.13 ± 0.20	(−0.33–0.59)	0.538

### Intraoperative Speech Errors

The superior frontal gyrus (SFG) was exposed in 21 (87.5%), the middle frontal gyrus (MFG) in 23 (95.8%), and the inferior frontal gyrus (IFG) in 23 (95.8%) craniotomies. A total of 33 cortical positive responses for language were recorded. Two (6.0%) responses were recorded in the SFG, 15 (45.5%) in the MFG, and 16 (48.5%) in the IFG. Of relevance is the fact that all positive responses recorded in the MFG were demonstrated in area 55b. The following responses were identified: speech arrest in 20 patients (13 in IFG; seven in area 55b), hesitation in one patient (area 55b), decreased fluency in one patient (SFG), phonemic errors in seven patients (four in area 55b and three in IFG), semantic paraphasias in two patients (area 55b), and anomia in two patients (one in MFG and one in SFG). At the subcortical level, 29 positive responses were identified and divided into two main areas: 21 patients in the deep IFG–MFG area and eight patients in the deep MFG–SFG area. These positive responses were divided as follows: repetition disturbance in one patient (deep IFG), speech arrest in one patient (deep IFG), hesitation in four patients (deep MFG–SFG), decreased fluency in three patients (deep SFG), phonemic errors in four patients (deep IFG–MFG), semantic paraphasias in seven patients (deep IFG–MFG), and nine anomias (eight in deep IFG–MFG and one in deep MFG–SFG) (Figure 2).

### Postoperative Language Assessment

Twenty (83.3%) patients had a transient deterioration of their language function after surgery (mean postoperative SST = 14.67 ± 0.76). Both the expressive (−2.875 ± 0.55) and the receptive (−1.36 ± 0.37) components of the SST deteriorated, with a statistically significant greater deterioration of the expressive component (*p* = 0.03). The single involvement of a particular gyrus (including area 55b) was not related *per se* with significant changes in language outcomes. The number of gyri with documented intraoperative positive language mapping was correlated with language outcomes: an increased number of gyri involvement was related with a better preoperative assessment (*p* = 0.037) and worse immediate language outcome (*p* = 0.029). This is mainly due to the changes in the expressive component of language (SST expressive preoperatively—*p* = 0.045) and SST expressive postoperatively−0.030). No significant changes were identified at the level of the receptive component of language. At the subcortical level, the involvement of the deep white matter of the SFG–MFG was related with worse expressive outcome postoperatively (*p* = 0.037), but no correlation was identified with the preoperative assessment (*p* = 0.780), and no overall impact was reflected in the overall SST assessment (SST preoperatively—*p* = 0.895; SST postoperatively−0.109). The preoperative nTMS mapping was not related with the language outcome (Table 4).

**Table 4:** Cortical and subcortical intraoperative involvement and pre- and postoperative language outcomes.

	**Coef**.	**95%CI**	***p*-value**
**Number of gyri involved**			
SST preoperative	1.35 ± 0.65	(0.08–2.62)	0.037
SST postoperative	−0.66 ± 0.30	(−1.26 to −0.69)	0.029
SST receptive preoperative	0.25 ± 0.21	(−0.20–0.69)	0.250
SST receptive postoperative	−0.11 ± 0.11	(−0.34–0.12)	0.316
SST expressive preoperative	0.59 ± 0.26	(0.01–1.16)	0.045
SST expressive postoperative	−0.17 ± 0.07	(−0.32 to −0.02)	0.030
**Subcortical involvement – SFG-MFG**			
SST preoperative	−0.06 ± 0.48	(−1.00–0.88)	0.895
SST postoperative	−0.31 ± 0.19	(−0.69–0.07)	0.109
SST receptive preoperative	−0.18 ± 0.61	(−1.36–1.01)	0.770
SST receptive postoperative	−0.41 ± 0.37	(−1.14–0.32)	0.276
SST expressive preoperative	0.32 ± 1.14	(−1.91–2.55)	0.780
SST expressive postoperative	−0.72 ± 0.34	(−1.40 to −0.04)	0.037
**Subcortical involvement – IFG-MFG**			
SST preoperative	0.29 ± 0.48	(−0.64–1.22)	0.544
SST postoperative	0.24 ± 0.18	(−0.12–0.60)	0.193
SST receptive preoperative	0.57 ± 0.62	(−0.64–1.78)	0.357
SST receptive postoperative	0.20 ± 0.33	(−0.46–0.85)	0.557
SST expressive preoperative	−0.01 ± 1.13	(−2.23–2.21)	0.992
SST expressive postoperative	0.66 ± 0.34	(−0.01–1.33)	0.055

## Discussion

The inferior frontal gyrus and the “classical” Broca's area have been traditionally considered as the main language hub in the dominant frontal lobe. However, the findings from multiple intraoperative reports showed that the functional organization of the frontal lobe is more complex, with positive language sites described both at the level of the MFG and, to a lesser extent, in the SFG (34–39).

In recent years, there has been a renewed interest in the role of the MFG as part of the language network. This is particularly the case after the description of area 55b, located at the posterior aspect of the MFG (7). Previous studies have implicated the role of posterior MFG and area 55b in language (7, 9, 10). In the present series, we report a high rate of positive speech responses at the level of the MFG (45% of intraoperative errors).

Two points require further discussion: First, the high incidence of positive responses in the MFG were replicated with the use of preoperative nTMS in addition to intraoperative DES. In addition, the majority of positive language sites in the MFG were confined to the posterior aspect of the gyrus, covering the anatomical location of area 55b. We hypothesize that these results can be explained due to the involvement of area 55b when stimulating the posterior aspect of the MFG. This is consistent with previous descriptions of the intraoperative responses obtained at area 55b (9).

Second, the responses recorded in the MFG with the combined nTMS and DES were both phonological (hesitations and phonemic errors) and semantic (semantic paraphasias and anomias). The involvement of this area in both semantic processing (12) and speech articulation (40) has been well recognized. The results therefore show that the posterior MFG is likely implicated in both the “dorsal phonological” and the “ventral semantic” streams of language (36). The involvement of the posterior MFG area in both streams of language was also supported at the subcortical level, where again both phonological (speech arrest, hesitation, and fluency disturbance) and semantic disturbances (semantic paraphasia and anomia) were elicited while stimulating the white matter deep to the MFG.

The subcortical areas were divided into two main areas (IFG–MFG and MFG–SFG) as the included tumors all involved more than one subcortical area. The majority of the recorded errors at the subcortical level occurred in the IFG–MFG area (72.4%). Both the arcuate fasciculus (AF) and the inferior fronto-occipital fasciculus (IFOF) are known to cross deep to the IFG–MFG area, with terminations at the level of the posterior MFG. AF, the main dorsal stream fasciculus, has terminations in the MFG documented by anatomical cadaveric, diffusion imaging, and resting-state fMRI studies. It is reported that up to 56% of patients can have terminations of the AF in the MFG, particularly the long segment (41–44). From a ventral stream perspective, multiple components of the IFOF were proposed based on anatomical studies (45), DES (46), and diffusion imaging (47). These methods concur that this tract has a termination in the posterior aspect of the MFG and therefore may serve as a substrate for the semantic errors identified in this area (45). The errors detected at the level of the MFG–SFG could be related to the stimulation of the fronto-aslant tract (hesitation and decreased fluency). This tract has been recently involved in the dorsal stream functions of language, particularly the fluency and initiation of speech (48–50).

In addition, the original structural connectivity data presented also support a strong connectivity of the MFG with the adjacent cortical gyri (IFG and SFG), likely mediated *via* U-fiber short association fibers. These findings are similar to those of other connectivity studies reported in the literature (7, 8). In this context, the interaction with the FEF in the anterior/middle frontal gyrus raises the possibility of a potential integration of visual recognition processes with speech production (51).

Therefore, two hypotheses can be formulated to support the interaction of posterior MFG and area 55b with both streams of language: a direct connectivity *via* relay of some subcomponents of both AF and IFOF and an indirect connectivity *via* the stimulation of U-fibers to the adjacent gyri (IFG and SMA). In addition to previous imaging and dissection data, recent nTMS data support a strong connectivity of language positive sites *via* the U-fiber system, supporting the indirect connectivity theory (52). The strong connectivity to the primary motor cortex further supports the potential role for hand movement integration in language (43) and the involvement of this area in the articulation and praxis of speech (9).

The impact of positive responses in the area 55b on clinical outcomes is difficult to establish, as these are usually associated with positive responses in either the IFG or SFG. Generally, the overall SST score and each of its components deteriorated temporarily after resection. Thus, the direct involvement of the posterior middle frontal gyrus and area 55b was not related with language outcome. However, the involvement of an increased number of gyri was related with better preoperative SST but with worse postoperative SST scores, particularly due to the receptive component. We believe that neuroplasticity within the language network can be partially responsible for these findings. A higher number of involved gyri may imply the involvement of preoperative adaptive mechanisms to maintain a high level of language function. However, at the same time, they may represent an overall stretched network that has a limited ability to recover from the hit provided by surgical resection and therefore linked to worse language outcomes. This natural process of adaptation has been seen in other systems of the human brain, such as the motor system (53), and further studies are required to ascertain if a similar process may be involved in the language connectivity and network.

There is evidence for language network plasticity in patients, given the intrinsic changes in the intra- and interhemispheric inhibition mechanisms altered by pathological condition*s* (54). Furthermore, multiple preoperative and intraoperative studies have documented the presence of language network plasticity, particularly in tumors with a long course and natural history, such as low-grade gliomas (55–59).

Despite it being acknowledged that there are different degrees of plasticity potential for different functions of language (60), we hypothesize that an increased number of frontal lobe gyri involved in language may act as a surrogate for the degree of plasticity and adaptation of the language network already present before surgery.

To this regard, preoperative speech mapping with nTMS can play an important role in detecting the extent of involvement of the different frontal gyri in language function, thus providing a useful tool for preoperative counseling. It is crucial to take a patient-centred approach in neuro-oncology in order to meet patient expectations with surgical and oncological treatment (61).

This study has the general limitations of a retrospective cohort study. The most significant one is the incomplete preoperative data for some of the patients included, where the posterior middle frontal gyrus and the area 55b were mapped intraoperatively. However, it provides evidence for the added value of the integration of preoperative advanced mapping and intraoperative language mapping of area 55b and further establishes this area within the MFG as a potential relay for both ventral and dorsal streams of language.

## Conclusion

This case series suggests that the posterior MFG, including area 55b, is an important integration cortical hub for both dorsal and ventral streams of language. It demonstrates this area as a cluster of positive responses in the MFG for both preoperative nTMS and intraoperative DES language mapping with a potential impact on language outcomes in dominant frontal lobe surgery.

## Data Availability Statement

The original contributions presented in the study are included in the article/Supplementary Material, further inquiries can be directed to the corresponding author/s.

## Ethics Statement

Written informed consent was obtained from the individual(s) for the publication of any potentially identifiable images or data included in this article.

## Author Contributions

SH and MA: data collection, data analysis, literature review, and manuscript writing. JL: data collection, data analysis, literature review, manuscript writing, and statistical analysis. SP: data collection (nTMS, demographics), and written part of methodology. HW: reviewed speech and language data, written part of methodology. OL, CS, HI, AM, and SO: area 55b seed, connectivity data. JL, AK-V, RG, KA, and RB: reviewed manuscript. FV: principal manuscript reviewer, innovative concepts and ideas. All authors contributed to the article and approved the submitted version.

## Conflict of Interest

The authors declare that the research was conducted in the absence of any commercial or financial relationships that could be construed as a potential conflict of interest.

## Supplementary Material

Supplementary Material 1 Tractography. Diffusion and T1-weighted data were pre-processed using the framework described by Mancini et al. (62). Multi-fiber orientations were estimated using single-shell two-tissue constrained spherical deconvolution using order l_max_ = 8 (63). Probabilistic tractography was done using Second-order integration over Fiber Orientation Distributions (iFOD2) (63) and seeding randomly from the white-matter/grey-matter interface, both to reconstruct whole-brain (FOD amplitude cut-off = 0.05 and total_streamlines = 10 million) and region-of-interest (ROI) based tractography (FOD amplitude cut-off = 0.05 and total_streamlines = 1,000). ROI-based tractography relied on reconstructing the arcuate fasciculus (AF) tract from the left side of the brain based on similar pipeline introduced in (62). The posterior MFG was used to filter the reconstructed AF and its connectivity analysis. Additionally, qualitative results are shown in Figure 3. For surgical planning purposes, region of interest based tractography using subcortical anatomical areas was performed as described by Fekonja et al., in (64). The nTMS responses were overlayed over the dissected tracts considered for language network.

Supplementary Video 1 Preoperative language mapping (nTMS assessment) demonstrating language errors.
