# Supplementary material for: Middle Frontal Gyrus and Area 55b: Perioperative Mapping and Language Outcomes
Source: Front Neurol. 2021 Mar 10;12:646075. doi: 10.3389/fneur.2021.646075 (PMC7988187; doi:10.3389/fneur.2021.646075)
Supplement: Supplementary Material 1 — Tractography. Diffusion and T1-weighted data were pre-processed using the framework described by Mancini et al. (62). Multi-fiber orientations were estimated using single-shell two-tissue constrained spherical deconvolution using order lmax = 8 (63). Probabilistic tractography was done using Second-order integration over Fiber Orientation Distributions (iFOD2) (63) and seeding randomly from the white-matter/grey-matter interface, both to reconstruct whole-brain (FOD amplitude cut-off = 0.05 and total_streamlines = 10 million) and region-of-interest (ROI) based tractography (FOD amplitude cut-off = 0.05 and total_streamlines = 1,000). ROI-based tractography relied on reconstructing the arcuate fasciculus (AF) tract from the left side of the brain based on similar pipeline introduced in (62). The posterior MFG was used to filter the reconstructed AF and its connectivity analysis. Additionally, qualitative results are shown in Figure 3. For surgical planning purposes, region of interest based tractography using subcortical anatomical areas was performed as described by Fekonja et al., in (64). The nTMS responses were overlayed over the dissected tracts considered for language network. [file Data_Sheet_1.docx]

*Tractography*

Diffusion and T1-weighted data were pre-processed using the framework described by Mancini et al. (Mancini et al., 2020). Multi-fibre orientations were estimated using single-shell two-tissue constrained spherical deconvolution using order l_max_=8 (Tournier et al., 2019). Probabilistic tractography was done using Second-order integration over Fibre Orientation Distributions (iFOD2) (Tournier et al., 2019) and seeding randomly from the white-matter/grey-matter interface, both to reconstruct whole-brain (FOD amplitude cut-off = 0.05 and total_streamlines = 10 million) and region-of-interest (ROI) based tractography (FOD amplitude cut-off = 0.05 and total_streamlines = 1000). ROI-based tractography relied on reconstructing the arcuate fasciculus (AF) tract from the left side of the brain based on similar pipeline introduced in (Mancini et al., 2020). The posterior MFG was used to filter the reconstructed AF and its connectivity analysis. Additionally, qualitative results are shown in **Figure 3.** For surgical planning purposes, region of interest based tractography using subcortical anatomical areas was performed as described by Fekonja et al., (2019). The nTMS responses were overlayed over the dissected tracts considered

for language network.
